# Generation of a Novel T Cell Specific Interleukin-1 Receptor Type 1 Conditional Knock Out Mouse Reveals Intrinsic Defects in Survival, Expansion and Cytokine Production of CD4 T Cells

**DOI:** 10.1371/journal.pone.0161505

**Published:** 2016-08-23

**Authors:** Ilgiz A. Mufazalov, Tommy Regen, Carsten Schelmbauer, Janina Kuschmann, Alisa M. Muratova, Alexei Nikolaev, Werner Müller, Emmanuel Pinteaux, Ari Waisman

**Affiliations:** 1 Institute for Molecular Medicine, University Medical Center of the Johannes Gutenberg-University Mainz, Mainz, Germany; 2 Department of Immunology, Faculty of Biology, Lomonosov Moscow State University, Moscow, Russia; 3 Faculty of Biology, Medicine and Health University of Manchester, Manchester, United Kingdom; University of Lisbon, PORTUGAL

## Abstract

Interleukin-1 (IL-1) plays a crucial role in numerous inflammatory diseases via action on its only known signaling IL-1 receptor type 1 (IL-1R1). To investigate the role of IL-1 signaling in selected cell types, we generated a new mouse strain in which exon 5 of the *Il1r1* gene is flanked by loxP sites. Crossing of these mice with *CD4-Cre* transgenic mice resulted in IL-1R1 loss of function specifically in T cells. These mice, termed IL-1R1^ΔT^, displayed normal development under steady state conditions. Importantly, isolated CD4 positive T cells retained their capacity to differentiate toward Th1 or Th17 cell lineages *in vitro*, and strongly proliferated in cultures supplemented with either anti-CD3/CD28 or Concanavalin A, but, as predicted, were completely unresponsive to IL-1β administration. Furthermore, IL-1R1^ΔT^ mice were protected from gut inflammation in the anti-CD3 treatment model, due to dramatically reduced frequencies and absolute numbers of IL-17A and interferon (IFN)-γ producing cells. Taken together, our data shows the necessity of intact IL-1 signaling for survival and expansion of CD4 T cells that were developed in an otherwise IL-1 sufficient environment.

## Introduction

Interleukin-1 (IL-1) is a pro-inflammatory cytokine that plays a prominent role in inflammation and immunoregulation. Its importance was confirmed by a variety of drugs designed to neutralize IL-1 activity, that showed beneficial effects in clinical settings [1]. Despite the complex process of IL-1 maturation and secretion [1, 2], its biological activity is also tightly controlled by numerous negative signaling regulators. For instance, IL-1 receptor antagonist competes with IL-1 for its signaling IL-1 receptor type 1 (IL-1R1). The soluble form of IL-1R1, as well as the soluble and membrane-bound IL-1 receptor type 2 (IL-1R2), can bind the cytokine and reduce its circulating levels. Furthermore, an intracellular form of IL-1R2 can be negatively involved in the regulation of IL-1 maturation [3]. And only the association of IL-1 with membrane-bound IL-1R1 leads to signal transduction and activation of NF-κB genes depending on the physiological context [4].

Since gene-targeting technology was established, our knowledge about the impact of different factors on physiology and pathophysiology of diseases has greatly increased. Early independent studies showed that mice deficient for IL-1 signaling, i.e. mice deficient for both IL-1α and IL-1β or for IL-1R1, develop normally under steady state conditions [5–7]. However, when challenged with pathogens or upon immunization, these animals showed a strong phenotype pointing to an essential role for IL-1 signaling in inflammation. Thus, IL-1R1 deficient mice were shown to be highly susceptible to *L*. *monocytogenes* [7], *C*. *rodentium* [8], and, in contrast, were resistant to autoimmune disease models, such as experimental autoimmune myocarditis [9] and experimental autoimmune encephalomyelitis, a model for multiple sclerosis [10, 11].

Importantly, a variety of immune and non-immune cell types express IL-1R1 making interpretation of the aforementioned data difficult. Moreover, although IL-1R1 expression is not abundant on the cell surface, only a few ligand-occupied receptors per cell are already sufficient to induce a strong response [4].

In order to study cell-type specific functions of IL-1 signaling, it is crucial to develop a system with conditional deletion of IL-1R1. Furthermore, to completely inactivate IL-1 signaling, it is necessary to delete both known isoforms of IL-1R1, as a short form that lacks the first three exons might retain signaling capacity, as was shown for the previously generated *Il1r1* knock out mice [12]. Here, we report a unique system of genetically modified mice in which exon 5 of the *Il1r1* gene was selectively deleted in T cells by using mice carrying a novel *Il1r1* conditional allele crossed to the *CD4-Cre* line. Comprehensive analysis of mutant mice confirmed a redundant function of IL-1 signaling for T cell development under steady state conditions and revealed its requirement when mice were challenged with CD3 specific antibodies. In this *in vivo* model, IL-1R1 deficient CD4 T cells were impaired in expansion and cytokine production. Thus, T cell specific IL-1R1 deficient animals represent a new tool to study the effects of IL-1 signaling on T cell functions.

## Material and Methods

### Mice, CFA Immunization and Anti-CD3 Treatment

*Il1r1*^fl/fl^, *CD4-Cre* and *CMV-Cre* mice were bred in-house under SPF conditions. Age and gender matched genetically modified animals carrying loxP sites without *Cre* transgene were considered as controls. All experiments were performed with 7–16 week old mice (unless otherwise specified) on C57BL/6 background in accordance with the guidelines of the Central Animal Facility Institution (CLAF, University of Mainz). Animal Care and Use Committee (IACUC) from the Land of Rhineland Palatine (RLP) approved all experiments with Permit Number 23 177-07/G12-1-057. Mice were euthanized with an overdose of isoflurane.

For the analysis of IL-1R1 expression, mice were immunized subcutaneously at the base of the tail with 100 μl of Complete Freund's Adjuvant (CFA, Difco) and were sacrificed 5 days post immunization.

Anti-CD3 treatment was performed by repetitive intraperitoneal injections of 20 μg mitogenic CD3-specific antibodies (BioXCell) every 48 h [13]. Mice were sacrificed and analysed at 48 h and 100 h after the first injection. Small intestine lamina propria (LPL) and intraepithelial (IEL) lymphocytes were isolated by using a combination of mechanical dissociation and enzymatic digestion with subsequent Percoll (Sigma) gradient separation as previously described [14].

### Generation of IL-1R1^ΔT^ and IL-1R1^-/-^ Mice

Generation of *Il1r1*^fl/fl^ mice with exon 5 of *Il1r1* gene flanked by loxP sites are described in details elsewhere [15]. To obtain *Il1r1* deletion specifically in αβ TCR^+^ T cells, *Il1r1*^fl/fl^ mice were crossed to *CD4-Cre* transgenic mice [16] resulting in the IL-1R1^ΔT^ mouse strain. For germline deletion *Il1r1*^fl/fl^ mice were crossed to *CMV-Cre* transgenic mice [17] resulting in a new IL-1R1^-/-^ mouse strain.

### Cytokines and Cell Culture

CD4 T cells were isolated from spleen and lymph nodes by MACS purification (Miltenyi), according to the manufacturer’s recommendations.

For *in vitro* proliferation and survival assay cells were labeled by using CellTrace violet cell proliferation kit (Invitrogen) according to the manufacturer’s recommendations and thereafter cultured at a concentration of 1.5×10^5^ cells/well in 200 μl T cell medium (RPMI medium supplemented with 10% FCS, 2mM L-glutamine, 100 units/ml penicillin, 100 mg/mL streptomycin, 1 mM sodium pyruvate, 50 mM 2-mercaptoethanol, 10 mM HEPES and 1% non-essential amino acids) for 4 days in 96-well plates as triplicates and pooled before the analysis. For cell stimulation, 1 μg/ml α-CD3, 6 ng/ml α-CD28 antibodies (BioXCell), 4 ng/ml IL-1β (R&D Systems), 1 μg/ml Concanavalin A (Con A, Sigma) were used.

For *in vitro* polarization, cells were cultured at a concentration of 2.0×10^5^ cells/well in 200 μl T cell medium for 4 days in 96-well plates as triplicates and pooled before the analysis. Cells were stimulated in the presence of 1 μg/ml α-CD3 and 6 ng/ml α-CD28 –Th0 condition; or in the presence of 1 μg/ml α-CD3, 6 ng/ml α-CD28, 2 ng/ml TGFβ (R&D Systems), 10 ng/ml IL-6 (Promocell), 10 ng/ml IL-23 (Miltenyi) and 2.5 μg/ml α-IFNγ (BioXCell)–Th17 condition; or in the presence of 1 μg/ml α-CD3, 6 ng/ml α-CD28, 4 ng/ml IL-12 (Promocell), 10 ng/ml IL-2 (Promocell) and 100 ng/ml IFNγ (R&D Systems)–Th1 condition.

### Antibodies and Flow Cytometry

Cells were surface stained with antibodies specific to γδ-TCR, CD25 (BioLegend), CD8, CD44, CD45.2, CD90.2 (BioLegend, eBioscience), TCR-β, CD4 (BioLegend, BD Biosciences), B220 (BioLegend, Invitrogen) and anti-CD62L (Immunotools). To exclude dead cells samples were stained with fixable (eBioscience) and non-fixable (Invitrogen) viability dyes (VD) together with surface antibodies. Antibodies detecting IFNγ, FoxP3 (eBioscience), IL-17A (eBioscience, BD Biosciences) and IL-1R1 (BioLegend) were applied after fixation and permeabilization of cells with Cytofix/Cytoperm (BD Biosciences) or FoxP3 staining kit (eBiosciences) according to the manufacturer’s recommendations. To detect cytokines, cells were restimulated for 4 h at (4–20)×10^6^ cells/ml in T cell medium using a combination of 50 ng/ml Phorbol-12-myristate-13-acetate (PMA, Sigma) and 500 ng/ml ionomycin (Invitrogen) in the presence of 1 μg/ml Brefeldin A (AppliChem).

Stained cells were acquired on FACS-Canto II (BD Biosciences) and were further analyzed with FlowJo software.

### Immunohistochemistry

For immunohistochemistry, small intestine (ileum) samples were isolated from naive, 9-week old mice. Immunofluorescence of cryo-sections was performed using the TSA Cy3 and Fluorescein systems (PerkinElmer). In brief, samples were stained at +4°C in a humid chamber with primary antibodies against CD4 (BD Bioscience, 553043). Slides were then incubated for 30 minutes at room temperature with biotinylated secondary antibodies (anti-Rat, BD Bioscience, 554014). Next, samples were stained with antibodies against TCRβ (BD Bioscience, 553169). Nuclei were counterstained with DAPI (Vector Laboratories, Ltd). Stained sections were visualized using a fluorescence microscope (Olympus IX81).

### Statistical Analysis

Graphical and statistical analysis was done using Prism 5 software (GraphPhad). Statistical significance was calculated using the two-tailed unpaired t-test. Values of p < 0.05, p < 0.01, p < 0.001, were marked as *, **, and ***, respectively. N.S.–not significant.

## Results and Discussion

### Generation of the IL-1R1^ΔT^ Mouse Strain

**Fig 1:**
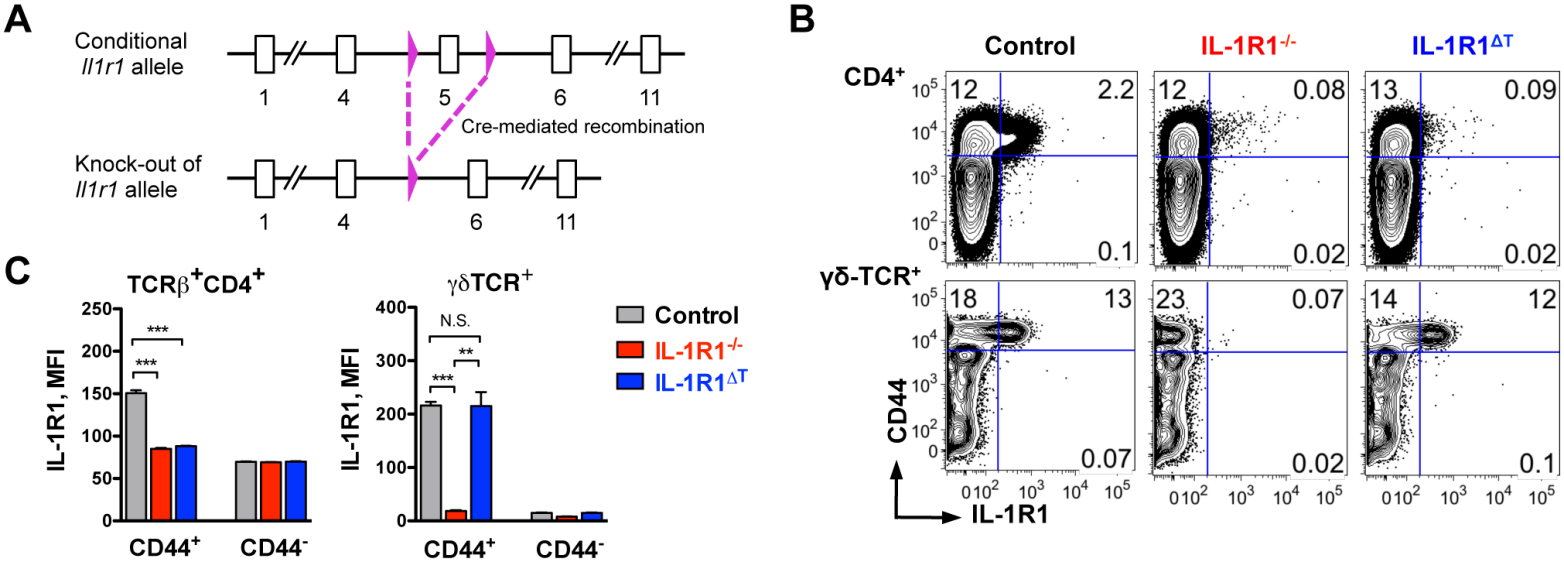
Conditional deletion of IL-1R1 in T cells. (A) Schematic representation of targeted murine *Il1r1* allele with exon 5 flanked by loxP sites. Cre mediated recombination results in excision of exon 5 and leads to the deletion of *Il1r1* gene. Open squares—numbered exons, triangles—loxP sites. All components are out of scale. (B) FACS analysis of IL-1R1 expression by CD4 T cells (upper row, gated on VD^-^/TCR-β^+^/CD4^+^ cells) and γδ-TCR^+^ T cells (lower row, gated on VD^-^/γδ-TCR^+^ cells) isolated from the draining lymph nodes of mice immunized with CFA. (C) Mean Fluorescence Intensity (MFI) of IL-1R1 staining by cell populations shown in (B). Data are (B) representative FACS plots and (C) mean +SEM of n = 3 of each genotype, and are representative of three independent experiments. **p < 0.01, ***p < 0.001, N. S.–not significant; two-tailed unpaired t-test.

IL-1α and IL-1β are cytokines with pleiotropic functions, which signal via the same receptor, IL-1R1. Although experiments using mice with a complete knock out of IL-1R1 helped in deciphering the role of IL-1 signaling especially in infection models and models of autoimmune diseases, those experiments had limitations when trying to evaluate the cell type specific contribution of IL-1 signaling in complex pathologies. To overcome this problem, we generated a new mouse strain that allows for Cre-recombinase mediated deletion of exon 5 of the *Il1r1* gene, leading to a transcriptional frame shift, which results in the inactivation of this gene (Fig 1A). When we analyzed IL-1R1^-/-^ mice in which exon 5 of the *Il1r1* gene was deleted in the germline by using a *CMV-Cre* deleter, we found that both αβ TCR^+^ CD4 T cells as well as γδ TCR^+^ T cells showed no IL-1R1 expression, as compared to similar T cell populations isolated from control animals (Fig 1B, left and middle). Next, we crossed mice with the conditional *Il1r1* allele to *CD4-Cre* mice, leading to the generation of a new mouse strain herein termed IL-1R1^ΔT^. As expected, IL-1R1^ΔT^ mice showed deletion of the receptor only in αβ TCR^+^ T cells but not in γδ TCR^+^ T cells, which are not affected, as they do not go through a stage of CD4 expression during their development (Fig 1B, right).

Importantly, IL-1R1 was exclusively expressed by T cells positive for the activation marker CD44, but not in a population negative for this glycoprotein. The mean fluorescence intensity (MFI) of IL-1R1 staining in CD44^+^ CD4 T cells isolated from either IL-1R1^ΔT^ or IL-1R1^-/-^ mice showed the same low levels as the CD44 negative population regardless of the genotype (Fig 1C, left). Equal to background levels of IL-1R1 MFI were also noted in CD44^+^ γδ T cells isolated from IL-1R1^-/-^ mice, but not in IL-1R1^ΔT^ mice (Fig 1C, right). Therefore, we confirmed deletion of IL-1R1 specifically in αβ TCR^+^ T cells of the IL-1R1^ΔT^ mice with unchanged receptor levels in non-αβ TCR^+^ T cells.

### CD4 T Cells Isolated from IL-1R1^ΔT^ Mice Do Not Respond to IL-1β *In Vitro*

**Fig 2:**
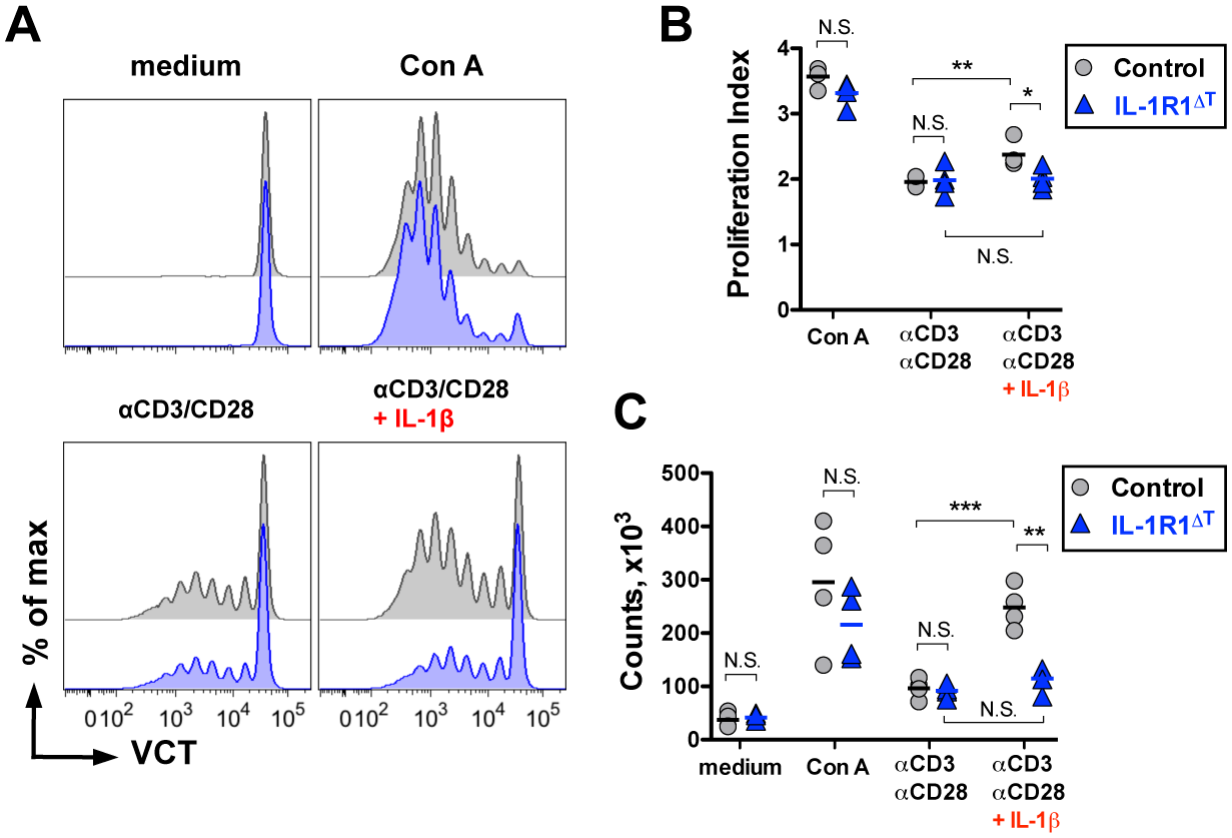
CD4 T cells isolated from IL-1R1^ΔT^ mice do not respond to IL-1β administration *in vitro*. (A) *In vitro* proliferation assay of CD4 T cells labeled with violet cell tracer (VCT) and activated with the indicated stimuli (FACS histograms, gated on VD^-^/CD4^+^ cells). (B) Proliferation index of cultures supplemented with indicated stimuli as shown in (A). (C) Total numbers of live CD4 T cells harvested from cultures supplemented with indicated stimuli as shown in (A). MACS-purified CD4 T cells from control and IL-1R1^ΔT^ mice (n = 4 of each genotype) were cultured 4 days under different indicated conditions. Data are (A) representative FACS histograms of control (black) and IL-1R1 deficient (blue) cultures and (B, C) individual values with mean. Experiments were performed twice with the similar results. *p < 0.05, **p < 0.01, ***p < 0.001, N. S.–not significant; two-tailed unpaired t-test.

To show the functional deletion of IL-1R1, we analyzed CD4 T cells isolated from IL-1R1^ΔT^ mice. In *in vitro* assays, CD4 T cells originating from IL-1R1^ΔT^ mice showed similar proliferation capacities as compared to T cells isolated from control animals, when triggered with either anti-CD3/CD28 antibodies or Con A (Fig 2A and 2B). In contrast, addition of IL-1β enhanced proliferation of the control T cells, but had no effect on cells lacking the receptor for this cytokine (Fig 2A and 2B). Similar changes could be noted when we calculated the absolute numbers of T cells in the cultures (Fig 2C). The observed differences in cell numbers likely reflect the reduced proliferation of mutant T cells. Taken together, these data confirm that IL-1 enhances proliferation of CD4 T cells, which is a known feature of the cytokine [18], and in addition, that our targeted mutation indeed abrogated the capacity of the T cells to respond to IL-1.

### Characterization of IL-1R1^ΔT^ Mice

**Fig 3:**
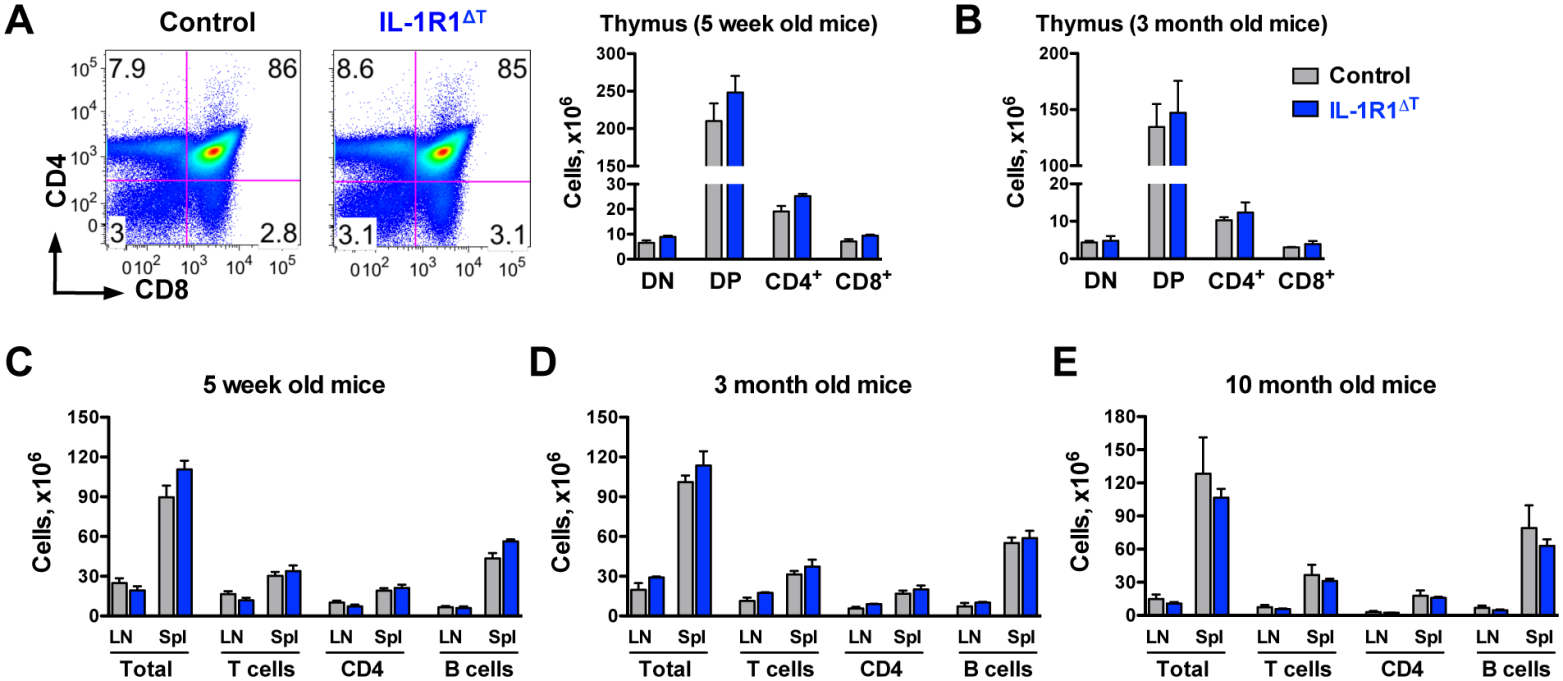
Normal T and B cell development in IL-1R1^ΔT^ mice. (A-B) Analysis of CD4 and CD8 T cells development in the thymus of (A) young and (B) adult mice. Data are (A) representative FACS plots gated on VD^-^/CD45.2^+^ cells and mean +SEM of n = 4 control, n = 3 IL-1R1^ΔT^, and (B) mean + SEM of n = 3 of each genotype. DN—CD4/CD8 double negative, DP—CD4/CD8 double positive cells. (C-E) Total cellularity, numbers of T cells, CD4 T cells and B cells isolated from the spleen and lymph nodes of (C) young, (D) adult and (E) aged mice. T cells (C, D) were defined as CD90.2^+^/TCR-β^+^ cells and (E) as CD90.2^+^ cells. B cells were defined as B220^+^ cells. Data are mean +SEM of (C) n = 5, (D) n = 3 of each genotype and of (E) n = 5 control, n = 3 IL-1R1^ΔT^ mice. LN—pooled inguinal, axillar and brachial lymph nodes, Spl—spleen. Experiments were performed at least twice with similar results.

Next, we analyzed whether αβ-TCR^+^ T cell specific deletion of IL-1R1 affects lymphocyte homeostasis. During development, immature αβ T cells pass through a CD4/CD8 double positive stage in the thymus. At this stage the *CD4-Cre* transgene is initially expressed leading to loxP/Cre mediated gene inactivation and subsequent generation of mature CD4 and CD8 single positive cells with the designed mutation. When we analysed thymocyte development of IL-1R1^ΔT^ mice we did not observe any differences, neither in early (CD4/CD8 double negative stage, when the *Cre* transgene is not yet expressed) nor late stages of αβ T cell development (Fig 3A and 3B). We also could not detect any changes in total cellularity, numbers of T cells, CD4 T cells and B220^+^ B cells isolated from secondary lymphoid organs of young, adult and aged IL-1R1^ΔT^ mice when compared to controls (Fig 3C, 3D and 3E). Together these data suggest a redundant function of IL-1 signaling in T cells in lymphocyte homeostasis of mice kept under SPF conditions.

**Fig 4:**
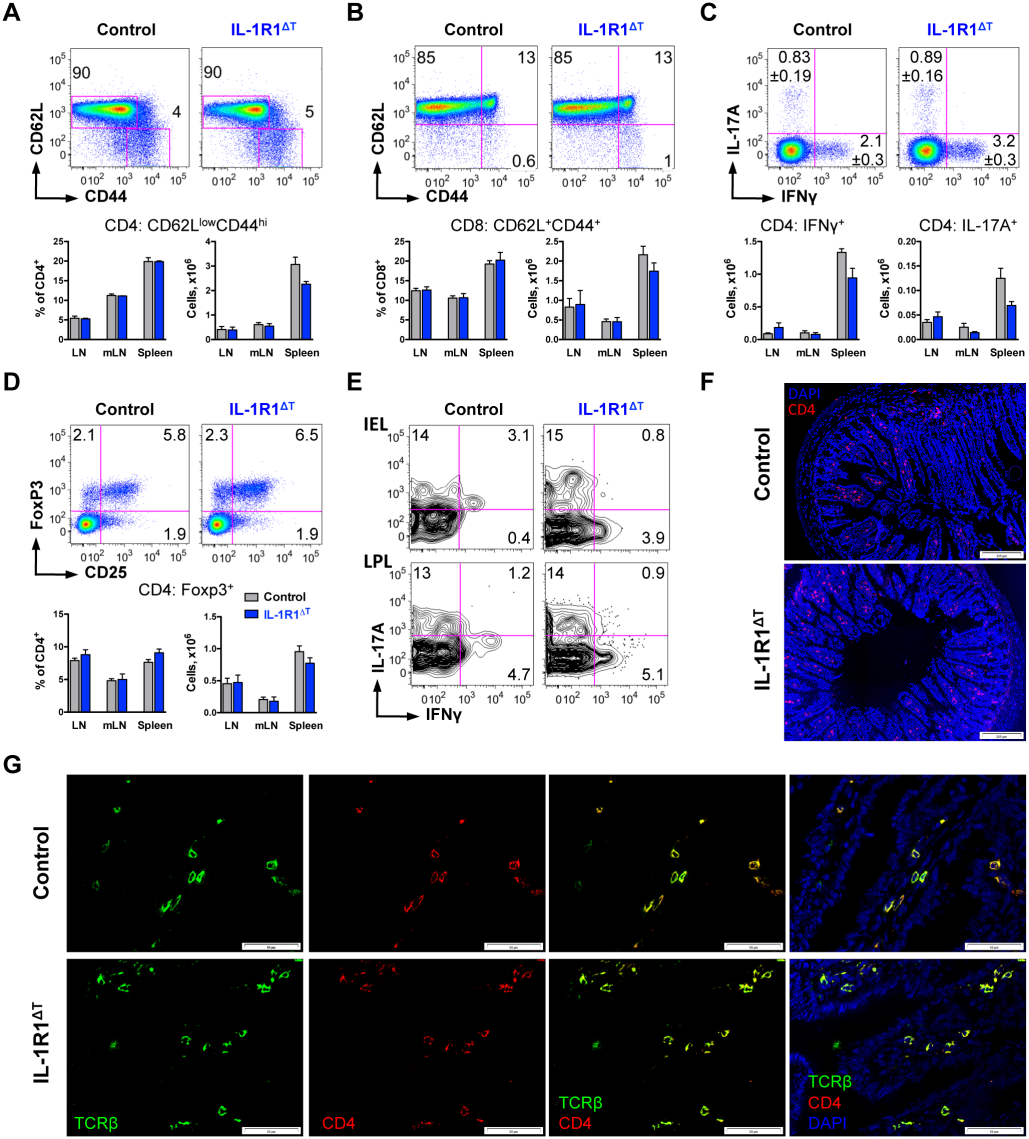
Normal CD4 and CD8 T cell development in IL-1R1^ΔT^ mice under steady state conditions. (A) Analysis of the activation status of CD4 T cells isolated from LN (FACS plots, gated on VD^-^/CD90.2^+^/CD4^+^ cells), mLN and the spleen. (B) Analysis of the activation status of CD8 T cells isolated from LN (FACS plots, gated on VD^-^/CD90.2^+^/CD8^+^ cells), mLN and the spleen. (C) Analysis of cytokine expression by CD4 T cells isolated from LN (FACS plots, gated on VD^-^/CD4^+^ cells), mLN and the spleen. (D) Analysis of FoxP3 and CD25 expression by CD4 T cells isolated from LN (FACS plots, gated on VD^-^/CD4^+^ cells), mLN and the spleen. Data (A-D) are shown as representative FACS plots with (C, D) mean frequencies per group and (C) indicated average deviation; and as bar diagram with mean +SEM. Data consist of (A, B, D) n = 6 control, n = 3 IL-1R1^ΔT^, and of (C) n = 3 of each genotype 3-month old mice. LN—pooled inguinal, axillar and brachial lymph nodes, mLN—mesenteric lymph nodes. Experiments (A-D) were performed three times with similar results. (E) Analysis of cytokine expression by T cells isolated from the small intestine. Data represent FACS plots gated on VD^-^/TCR-β^+^ cells of n = 5 pooled samples of each genotype. IEL—intraepithelial lymphocytes, LPL—lamina propria lymphocytes. (F) Immunofluorescence analysis of the small intestine (blue, DAPI; red, anti-CD4). Scale bar represents 200 μm. (G) Immunofluorescence analysis of the small intestine (blue, DAPI; red, anti-CD4; green, anti-TCRβ). Scale bar represents 50 μm. Experiments (E-G) were performed twice with similar results.

To better characterize the activation status of T cells in naïve IL-1R1^ΔT^ mice we first investigated the surface expression of CD62L and CD44 on CD4 T cells, and found the percentage of activated/memory cells to be equal in the different peripheral immune organs of IL-1R1^ΔT^ and control mice (Fig 4A). We observed only a small reduction in numbers of CD4 T cells with activated/memory phenotype in the spleen of IL-1R1^ΔT^ mice, which is, however was not present in inguinal, brachial and mesenteric lymph nodes (Fig 4A). Next, we analysed CD8 T cells and also could not detect differences in their activation status in mice deficient for IL-1R1 (Fig 4B).

Differentiated CD4 T cells can be subdivided into several T helper (Th) subsets based on their cytokine expression. When analysed, we observed a minor but not significant reduction of both IFNγ expressing (Th1) and IL-17A expressing (Th17) cell numbers in the spleen of non-immunized IL-1R1^ΔT^ mice (Fig 4C). Analysis of regulatory T cells (Treg) expressing transcription factor FoxP3 in IL-1R1 deficient animals also did not reveal significant changes in mutant mice when compared to control animals (Fig 4D).

The gastro-intestinal tract represents one of the barrier organs in which immune cells interact with pathogens and commensal micro flora and IL-17A producing CD4 T cells are abundantly present in the gut samples [19]. Therefore we aimed to investigate cytokine production by T cells in the small intestine of IL-1R1^ΔT^ mice under steady-state conditions. Isolated intraepithelial and lamina propria lymphocytes (IEL and LPL, respectively) displayed equal IL-17A and IFNγ producing T cells in both groups of animals (Fig 4E). Moreover, we did not find differences in structures of the ileum or in distribution of TCRβ^+^ CD4 T cells in naïve IL-1R1^ΔT^ mice (Fig 4F and 4G).

Taken together, we noted only minor, if any, changes in different CD4 T cell subsets in naive IL-1R1^ΔT^ mice. These results suggest that IL-1 signaling has very limited effect on the development and differentiation of T cells in animals kept under SPF conditions, and indicate that IL-1R1^ΔT^ mice should be analyzed also under conditions of immunological challenge.

### IL-1R1^ΔT^ Mice Are Protected from Gut Inflammation in Anti-CD3 Treatment Model

**Fig 5:**
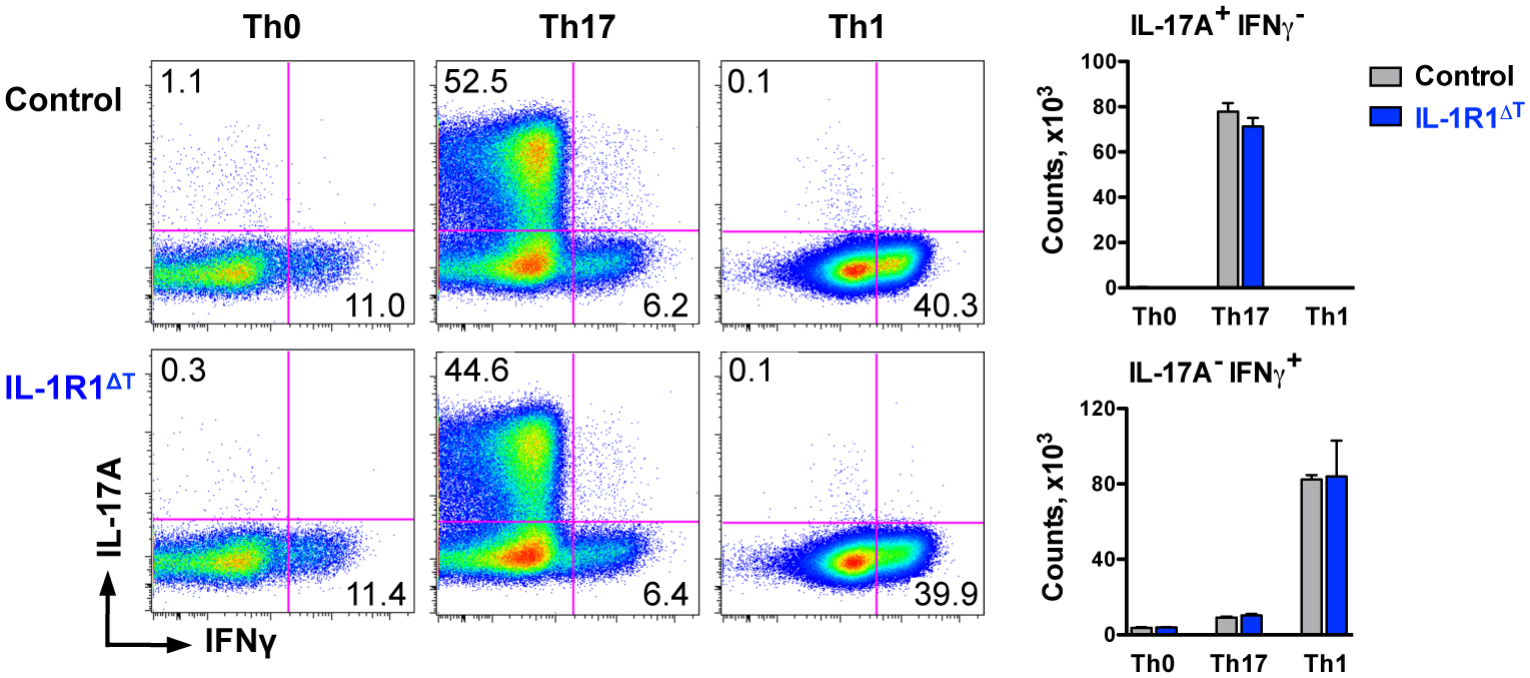
CD4 T cells isolated from IL-1R1^ΔT^ mice display non-impaired differentiation *in vitro*. *In vitro* polarization assay of MACS-purified CD4 T cells activated under Th0, Th17 and Th1 conditions (described in materials and methods section) for 4 days. Data are shown as representative FACS plots gated on VD^-^/CD4^+^ cells with average frequencies per group and as mean +SEM of n = 3 of each genotype. Experiments were performed twice with similar results.

**Fig 6:**
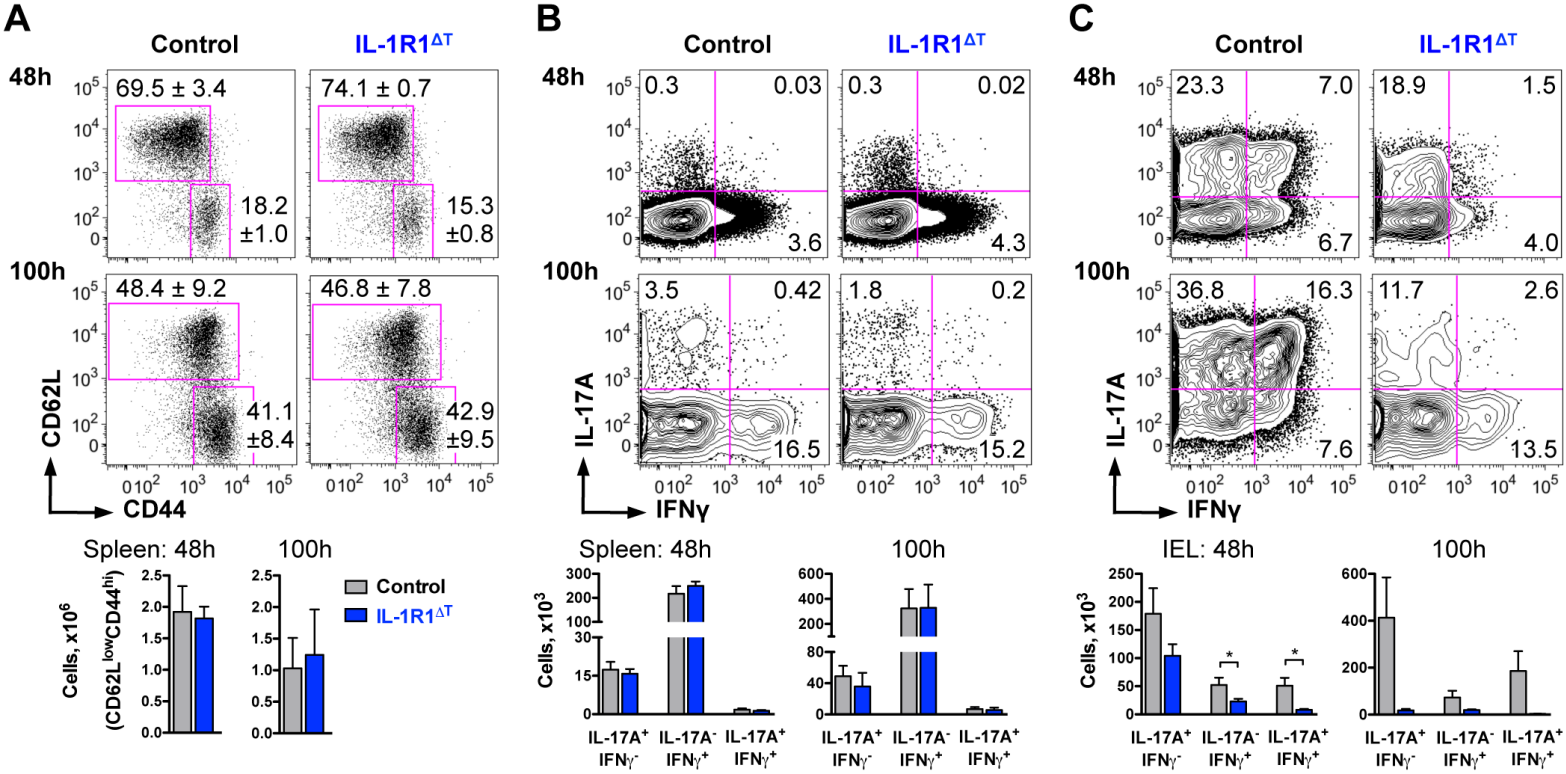
T cell specific deletion of IL-1R1 results in impaired Th17 cell expansion in anti-CD3 treatment model. (A) Analysis of activation status of CD4 T cells isolated from the spleen after anti-CD3 treatment at indicated time points. Data are shown as representative FACS plots gated on VD^-^/CD90.2^+^/CD4^+^ cells with mean frequencies per group ± average deviation; and as mean +SEM of n = 4 of each genotype. (B-C) Analysis of cytokine expression by CD4 T cells isolated from the (B) spleen and (C) IEL compartment of the small intestine after anti-CD3 treatment at indicated time points. Data (B, C) are shown as representative FACS plots gated on VD^-^/CD90.2^+^/CD4^+^ cells with mean frequencies per group and as mean +SEM of n = 4 control and n = 5 IL-1R1^ΔT^ mice analyzed at 48 h, as well as of n = 3 control and n = 4 IL1R1^ΔT^ mice analyzed at 100 h. Experiments were performed twice with similar results. *p < 0.05; two-tailed unpaired t-test.

In order to test how the lack of IL-1R1 specifically in T cells affects their *in vivo* development, we decided to use the model of anti-CD3 treatment. In this model, systemic anti-CD3 administration induces apoptosis of T cells and, subsequently, inflammation that results in the mobilization of effector T cells, mainly Th17 cells, from the peripheral immune organs to the gut [13]. Two cytokines essential for Th17 cell differentiation, namely TGFβ and IL-6, were shown to be strongly up-regulated after anti-CD3 treatment [13]. Therefore, at first we investigated the differentiation capacity of IL-1R1 deficient T cells *in vitro*. Both Th17 (driven by TGFβ, IL-6 and IL-23) and Th1 (driven by IL-12, IL-2 and IFNγ) polarization conditions led to equal responses of control and mutant cells when analyzed after 4 days of *in vitro* stimulation (Fig 5). Also taking into consideration the data from our previous *ex vivo* analysis (Fig 4C and 4E), we concluded that initial differentiation of mutant cells towards the Th17 and Th1 lineages is not affected by IL-1R1 deletion. To investigate Th17 cell development under inflammatory conditions *in vivo*, IL-1R1^ΔT^ mice as well as control mice were injected with anti-CD3 and were analyzed at two time points, 48 h and 100 h after immunization. The first time point should reveal the status of the remaining T cells (that escaped induced cell death) while the later time point should reflect the cells that already underwent proliferation. Such injections resulted in CD4 splenic T cell activation equally in control and IL-1R1^ΔT^ mice at early and later time points (Fig 6A). Moreover, as seen in Fig 6B, we did not observe major differences in the populations of CD4 T cells producing IFNγ, IL-17A or both cytokines in the spleen, neither after 48 h, nor after 100 h, when comparing IL-1R1^ΔT^ and control mice. These data suggest equal susceptibility of IL-1R1 deficient T cells to anti-CD3 induced cell death and unchanged development of both Th17 and Th1 cells in the periphery of IL-1R1^ΔT^ mice.

The main effect of anti-CD3 injection, however, can be seen in the gut, where Th17 cells home and proliferate extensively. To investigate whether IL-1 signaling has a role in this migration/proliferation, we also analyzed the intestinal intraepithelial lymphocytes. For that, we isolated the IELs from the small intestine of anti-CD3 injected mice, 48 and 100 h after injection. Already at the 48 h time point, we could detect a reduction in Th17 and Th1 cells in IL-1R1^ΔT^ mice, when compared to the control animals (Fig 6C). This was reflected by markedly decreased percentage of IL-17A/IFNγ double positive cells and total numbers of cytokine positive cells in the mutant mice. After 100 h we could not observe significant changes in frequencies of conventional Th1 cells, but rather saw a dramatic reduction of CD4 positive IL-17A producers, representing either Th17 cells or cells that produce also IFNγ (double producers, see Fig 6C). Importantly, the long-term (100 h) treatment resulted in an increased yield of Th17 cells in control mice, while the Th17 cell population in IL-1R1^ΔT^ mice remained largely unchanged, compared to the early (48 h) time point. These data are in line with the observation made *in vitro*, where IL-1R1 was found to be highly expressed in mouse Th17 cells [20], the deletion of which indeed abrogated the response to IL-1 in our study.

Thus, by using the anti-CD3 treatment model, we demonstrated CD4 T cell intrinsic requirements for intact IL-1 signaling for Th17 cell expansion *in vivo*. We also confirmed that IL-1R1^ΔT^ mice are fully functional and can be used in other models of immunological challenge. Finally, our data indicate that the anti-CD3 induced “cytokine storm” among other reported cytokines, such as TNF-α, IL-6, TGF-β [13, 21] should also contain IL-1 as we observed a clear IL-1R1-dependant effect on the gut inflammation.

Despite a tremendous amount of research on IL-1, neither the cytokine nor its receptor IL-1R1 was available as a mouse model with conditional deletion. It was only recently that we generated a microglia specific deletion of IL-1R1 [22]. In an independent study we also achieved the global deletion of IL-1R1 using the same *Il1r1* conditional allele. When these knock out mice were infected with helminth *T*. *muris* they could not clear the infection [15] resolving an old enigma of the resistance of previously generated IL-1R1 deficient mice to worm infection [23]. A possible explanation might be the residual activity of the remaining IL-1R1 isoform in the latter study, a disadvantage that was bypassed by deletion of exon 5 in our work [15]. IL-1R1 T cell specific knock out mice utilized in the present work were also fully functional and revealed an importance of intact intrinsic IL-1 signaling for T helper cell survival and expansion.

We believe that new mouse strains originating from our *Il1r1* floxed mice, together with the recently reported alternative *Il1r1* conditional knock out mice [24], which, however, were not yet tested in a cell-type specific manner, will provide useful tools to further investigate IL-1 functions.

## References

[1] Yazdi AS, Drexler SK. Regulation of interleukin 1alpha secretion by inflammasomes. Ann Rheum Dis. 2013;72 Suppl 2:ii96–9. Epub 2012/12/21. 10.1136/annrheumdis-2012-202252 . 23253918

[2] Lopez-Castejon G, Brough D. Understanding the mechanism of IL-1beta secretion. Cytokine Growth Factor Rev. 2011;22(4):189–95. Epub 2011/10/25. 10.1016/j.cytogfr.2011.10.001 22019906 PMC3714593

[3] Zheng Y, Humphry M, Maguire JJ, Bennett MR, Clarke MC. Intracellular interleukin-1 receptor 2 binding prevents cleavage and activity of interleukin-1alpha, controlling necrosis-induced sterile inflammation. Immunity. 2013;38(2):285–95. Epub 2013/02/12. 10.1016/j.immuni.2013.01.008 23395675 PMC3659285

[4] Subramaniam S, Stansberg C, Cunningham C. The interleukin 1 receptor family. Dev Comp Immunol. 2004;28(5):415–28. Epub 2004/04/06. 10.1016/j.dci.2003.09.016 . 15062641

[5] Glaccum MB, Stocking KL, Charrier K, Smith JL, Willis CR, Maliszewski C, et al Phenotypic and functional characterization of mice that lack the type I receptor for IL-1. J Immunol. 1997;159(7):3364–71. Epub 1997/10/08. . 9317135

[6] Horai R, Asano M, Sudo K, Kanuka H, Suzuki M, Nishihara M, et al Production of mice deficient in genes for interleukin (IL)-1alpha, IL-1beta, IL-1alpha/beta, and IL-1 receptor antagonist shows that IL-1beta is crucial in turpentine-induced fever development and glucocorticoid secretion. J Exp Med. 1998;187(9):1463–75. Epub 1998/06/06. 9565638 10.1084/jem.187.9.1463 PMC2212263

[7] Labow M, Shuster D, Zetterstrom M, Nunes P, Terry R, Cullinan EB, et al Absence of IL-1 signaling and reduced inflammatory response in IL-1 type I receptor-deficient mice. J Immunol. 1997;159(5):2452–61. Epub 1997/09/01. . 9278338

[8] Lebeis SL, Powell KR, Merlin D, Sherman MA, Kalman D. Interleukin-1 receptor signaling protects mice from lethal intestinal damage caused by the attaching and effacing pathogen Citrobacter rodentium. Infect Immun. 2009;77(2):604–14. Epub 2008/12/17. 10.1128/IAI.00907-08 19075023 PMC2632048

[9] Eriksson U, Kurrer MO, Sonderegger I, Iezzi G, Tafuri A, Hunziker L, et al Activation of dendritic cells through the interleukin 1 receptor 1 is critical for the induction of autoimmune myocarditis. J Exp Med. 2003;197(3):323–31. Epub 2003/02/05. 12566416 10.1084/jem.20021788 PMC2193833

[10] Matsuki T, Nakae S, Sudo K, Horai R, Iwakura Y. Abnormal T cell activation caused by the imbalance of the IL-1/IL-1R antagonist system is responsible for the development of experimental autoimmune encephalomyelitis. Int Immunol. 2006;18(2):399–407. Epub 2006/01/18. 10.1093/intimm/dxh379 . 16415102

[11] Sutton C, Brereton C, Keogh B, Mills KH, Lavelle EC. A crucial role for interleukin (IL)-1 in the induction of IL-17-producing T cells that mediate autoimmune encephalomyelitis. J Exp Med. 2006;203(7):1685–91. Epub 2006/07/05. 10.1084/jem.20060285 16818675 PMC2118338

[12] Qian J, Zhu L, Li Q, Belevych N, Chen Q, Zhao F, et al Interleukin-1R3 mediates interleukin-1-induced potassium current increase through fast activation of Akt kinase. Proc Natl Acad Sci U S A. 2012;109(30):12189–94. Epub 2012/07/11. 10.1073/pnas.1205207109 22778412 PMC3409776

[13] Esplugues E, Huber S, Gagliani N, Hauser AE, Town T, Wan YY, et al Control of TH17 cells occurs in the small intestine. Nature. 2011;475(7357):514–8. Epub 2011/07/19. 10.1038/nature10228 21765430 PMC3148838

[14] Reissig S, Hackenbruch C, Hovelmeyer N. Isolation of T cells from the gut. Methods Mol Biol. 2014;1193:21–5. Epub 2014/08/26. 10.1007/978-1-4939-1212-4_3 . 25150993

[15] Abdulaal WH, Walker CR, Costello R, Redondo-Castro E, Mufazalov IA, Papaemmanouil A, et al Characterization of a conditional interleukin-1 receptor 1 mouse mutant using the Cre/LoxP system. Eur J Immunol. 2016;46(4):912–8. 10.1002/eji.201546075 . 26692072 PMC4982085

[16] Lee PP, Fitzpatrick DR, Beard C, Jessup HK, Lehar S, Makar KW, et al A critical role for Dnmt1 and DNA methylation in T cell development, function, and survival. Immunity. 2001;15(5):763–74. Epub 2001/12/01. . 11728338 10.1016/s1074-7613(01)00227-8

[17] Schwenk F, Baron U, Rajewsky K. A cre-transgenic mouse strain for the ubiquitous deletion of loxP-flanked gene segments including deletion in germ cells. Nucleic Acids Res. 1995;23(24):5080–1. Epub 1995/12/25. 8559668 10.1093/nar/23.24.5080 PMC307516

[18] Ben-Sasson SZ, Hu-Li J, Quiel J, Cauchetaux S, Ratner M, Shapira I, et al IL-1 acts directly on CD4 T cells to enhance their antigen-driven expansion and differentiation. Proc Natl Acad Sci U S A. 2009;106(17):7119–24. 10.1073/pnas.0902745106. ISI:000265584500046. 19359475 PMC2678417

[19] Ivanov II, Atarashi K, Manel N, Brodie EL, Shima T, Karaoz U, et al Induction of Intestinal Th17 Cells by Segmented Filamentous Bacteria. Cell. 2009;139(3):485–98. 10.1016/j.cell.2009.09.033. ISI:000271259600014. 19836068 PMC2796826

[20] Guo L, Wei G, Zhu J, Liao W, Leonard WJ, Zhao K, et al IL-1 family members and STAT activators induce cytokine production by Th2, Th17, and Th1 cells. Proc Natl Acad Sci U S A. 2009;106(32):13463–8. Epub 2009/08/12. 10.1073/pnas.0906988106 19666510 PMC2726336

[21] Merger M, Viney JL, Borojevic R, Steele-Norwood D, Zhou P, Clark DA, et al Defining the roles of perforin, Fas/FasL, and tumour necrosis factor alpha in T cell induced mucosal damage in the mouse intestine. Gut. 2002;51(2):155–63. Epub 2002/07/16. 12117872 10.1136/gut.51.2.155 PMC1773316

[22] Bruttger J, Karram K, Wortge S, Regen T, Marini F, Hoppmann N, et al Genetic Cell Ablation Reveals Clusters of Local Self-Renewing Microglia in the Mammalian Central Nervous System. Immunity. 2015;43(1):92–106. Epub 2015/07/15. 10.1016/j.immuni.2015.06.012 . 26163371

[23] Humphreys NE, Grencis RK. IL-1-dependent, IL-1R1-independent resistance to gastrointestinal nematodes. Eur J Immunol. 2009;39(4):1036–45. 10.1002/eji.200838938. ISI:000265478700014. 19247983

[24] Robson MJ, Zhu CB, Quinlan MA, Botschner DA, Baganz NL, Lindler KM, et al Generation and Characterization of Mice Expressing a Conditional Allele of the Interleukin-1 Receptor Type 1. PloS one. 2016;11(3):e0150068 10.1371/journal.pone.0150068 26930558 PMC4773179

